# T-Cell/B-Cell Interactions in Atherosclerosis

**DOI:** 10.1161/ATVBAHA.124.319845

**Published:** 2024-05-30

**Authors:** Peter William Jones, Ziad Mallat, Meritxell Nus

**Affiliations:** 1 Cardiovascular Division, Department of Medicine, Heart and Lung Research Institute, University of Cambridge, United Kingdom (P.W.J., Z.M., M.N.).; 2 INSERM U970, Paris Cardiovascular Research Centre, France (Z.M.).

**Keywords:** adaptive immunity, atherosclerosis, B lymphocyte, humans, T lymphocytes

## Abstract

Atherosclerosis is a complex inflammatory disease in which the adaptive immune response plays an important role. While the overall impact of T and B cells in atherosclerosis is relatively well established, we are only beginning to understand how bidirectional T-cell/B-cell interactions can exert prominent atheroprotective and proatherogenic functions. In this review, we will focus on these T-cell/B-cell interactions and how we could use them to therapeutically target the adaptive immune response in atherosclerosis.

**Highlights**

- T cell–B cell interactions have a prominent role modulating atherosclerosis.
- T follicular helper cell–marginal zone B cell interaction protects from atherosclerosis.
- Cell-specific therapies targeting T cell–B cell interactions will be instrumental to modulate the development of atherosclerosis.

## ADAPTIVE IMMUNE RESPONSE AND ATHEROSCLEROSIS

Atherosclerosis is a complex inflammatory disease that is initiated by the accumulation of cholesterol-rich lipoproteins in the walls of large and medium-sized arteries and involves both vascular and immune cells. Both innate and adaptive immune responses have been causally involved in experimental atherogenesis. Recently, a few flagship clinical trials using anti-inflammatory drugs (ie, canakinumab) on top of lipid-lowering drugs have proven the inflammatory theory of atherosclerosis by reducing cardiovascular events.^1^ Unfortunately, dampening the immune response came with an increased incidence of fatal infection^2^; thus more selective immunotherapies are needed to treat atherosclerosis. Emerging evidence suggests that targeting the adaptive immune response (eg, with low-dose IL [interleukin]-2)^3^ could be a safe and effective option, and so the quest to find more selective immunotherapies for patients with atherosclerotic cardiovascular disease has begun.

**Please see www.ahajournals.org/atvb/atvb-focus for all articles published in this series.**

The adaptive immune response is a specialized response. Innate immune cells, mainly dendritic cells and macrophages, act as antigen-presenting cells and initiate the adaptive immune response. They drive the maturation and polarization of naive CD8^+^ or CD4^+^ T cells to specialized effector or memory cell subsets through recognizing antigenic peptides on MHC (major histocompatibility complex) class I or II molecules, engagement of costimulatory pathways, and the impact of microenvironmental cytokines at the site of antigen presentation (in secondary lymphoid organs, ie, draining lymph nodes and spleen, and locally in the atherosclerotic lesions, including the artery tertiary lymphoid organs [ATLOs]). The antigen epitopes that drive specific adaptive T-cell immune responses in atherosclerosis are still to be characterized. However, an increasing body of evidence suggests a potential role for native or oxidized ApoB100-derived epitopes.^4,5^ All types of CD4^+^ T cells have been found in atherosclerotic lesions and in the bloodstream of patients with atherosclerosis. There are at least 5 major T-cell subtypes involved in atherosclerosis, including effector T helper (T_H_) cells and T regulatory (T_REG_) cells. While T_REGS_ have been shown to be atheroprotective, T_H_ cells exhibit different roles depending on the subtype.^6^ T_H_1 are atherogenic cells, while T_H_2, T_H_17, and T follicular helper (T_FH_) cells may play context-dependent atherogenic or atheroprotective roles. One of the main functions of T cells is to regulate the B-cell adaptive humoral response and, as we know that this is directly regulated by T_FH_ cells, they will be the main subject for discussion in our review.

B cells can secrete antibodies in a T cell–dependent or T cell–independent manner. On one hand, there are B1 cells located in serous membrane cavities (ie, peritoneum, pleura, pericardium) that secrete natural IgM antibodies, have an atheroprotective role in mice,^7^ and their human counterparts are starting to be identified.^8^ On the other hand, B2 cells can be divided into atheroprotective marginal zone B (MZB) cells^9^ and proatherogenic follicular B cells.^10^ B2 cells participate in T cell–dependent responses, enter germinal center (GC) reactions, and undergo class switch recombination to produce immunoglobulin IgA, IgG, and IgE antibodies. Most studies indicate that GCs^11–13^ and their products, class-switched IgG^10,11^ and IgE,^14^ are proatherogenic in mice. This is thought to be due to the capacity of IgG and IgE to form immune complexes that cross-link activator Fc receptors and trigger vascular inflammation.^14–16^ Despite being overall proatherogenic,^17,18^ IgG autoantibodies to particular self-antigens may also have atheroprotective properties^19^ as is the case for anti-malondialdehyde-LDL IgG raised through vaccination.^20^ On the other hand, natural IgM antibodies coming from B1 cells,^7^ and T cell–dependent extrafollicular^21,22^ IgM antibodies^23,24^ coming from MZB cells^9,25^ protect from atherosclerosis. This is largely attributed to oxidation-specific epitope (OSE)–binding IgM antibodies that reduce inflammation within plaques by clearing apoptotic cells^26,27^ and neutralizing oxidized low-density lipoprotein (oxLDL) uptake.^28^ Reflecting this, several clinical studies indicate that oxLDL-specific IgM titers^29^ and even circulating MZB-like cell frequencies^30^ in patient blood inversely correlate with coronary artery disease severity while no consistent correlation has been reported with oxLDL-specific IgG.

T-cell/B-cell interactions are bidirectional, so not only do T cells modulate B-cell antibody secretion but B cells also modulate T-cell activation and differentiation. The importance of T-cell/B-cell interactions in atherosclerosis has been demonstrated using atherosclerotic mouse models. Combined B- and T-cell deficiency in atherosclerotic mice with deletion of recombination-activating genes (*Rag1* and *Rag2*) reduces early plaque development under moderate hypercholesterolemia.^31^ While this could be simply additive, or attributed to particular subsets, it is likely that T-cell/B-cell interactions contribute to this phenotype. Selective B-cell depletion with an anti-CD20 antibody^32^ and congenitally B-cell deficiency in μMT mice^33^ reduces atherosclerosis at least, in part, due to reduced T-cell activation and infiltration into plaques. Ait-Oufella et al^32^ showed that B-cell depletion enhanced T_H_17 polarization while decreasing proatherogenic T_H_1 cells and this atheroprotective effect could be abrogated by neutralizing the T_H_17 cytokine IL-17. Furthermore, Tay et al^33^ showed that T cells from congenitally B cell–deficient μMT mice with atherosclerosis have decreased proliferative capacity to in vitro oxLDL stimulation, perhaps owed to lack of previous B-cell antigen presentation.^34^ Moreover, pan-CD4^+^ T-cell depletion was found to decrease OSE-specific IgM, IgG1, as well as IgG2c and increased atherosclerosis, arguing for a potential role of T cells not only modulating GC and IgG secretion but also IgM secretion by B cells.^35^ While this initial research highlights the importance of CD4^+^ T-cell subsets and antibody-independent B-cell functions, most T-cell/B-cell interactions are thought to involve T_FH_ cells, which are specialized to promote antibody responses.

## T_FH_ CELLS IN ATHEROSCLEROSIS

T_FH_ cells express the signature transcription factor BCL6,^36,37^ and their polarization appears to be driven by IL-6 exposure in mice but IL-12, TGFβ (transforming growth factor-β), and activin A in humans.^38^ However, it is not yet well understood how naive CD4^+^ T cells commit to CXCR5^+^ pre-T_FH_ cells. Nonetheless, when a pre-T_FH_ located in the T cell zone of a secondary lymphoid organ is primed by a dendritic cell presenting antigen on MHCII, it will downregulate CCR7 and upregulate CXCR5 and migrate toward the border of B-cell follicles where it interacts with matching antigen specificity activated B cells.^39^ This second encounter with an antigen-presenting cell will induce pre-T_FH_ expression of costimulatory (eg, inducible costimulator [ICOS], CD40L) and coinhibitory (eg, PD1) molecules, as well as the secretion of signature cytokines (IL-21 and IL-4) to become mature T_FH_ cells. At this point, the T_FH_-cell/B2-cell interaction can initiate an (1) extrafollicular response where activated B cells become short-lived IgM-secreting plasmablasts^40^ or (2) GC response that leads to B-cell survival and thereby selection, affinity maturation, class switching (from IgM/IgD into IgG, IgE, or IgA), as well as differentiation into long-lived memory B cells and antibody-secreting plasma cells (PCs). As we will elaborate below, in atherosclerosis, T_FH_-MZB interactions at the T-B border are necessary for the formation of extrafollicular anti-OSE IgM-producing plasmablasts and for the complete differentiation of pre-T_FH_ to generate the GC response (Figure). Others have also found T_FH_ in atherosclerosis can derive from exT_REG_s, and, while the exact mechanism is not yet known, this conversion is prevented by apoAI.^41^

**Figure.**
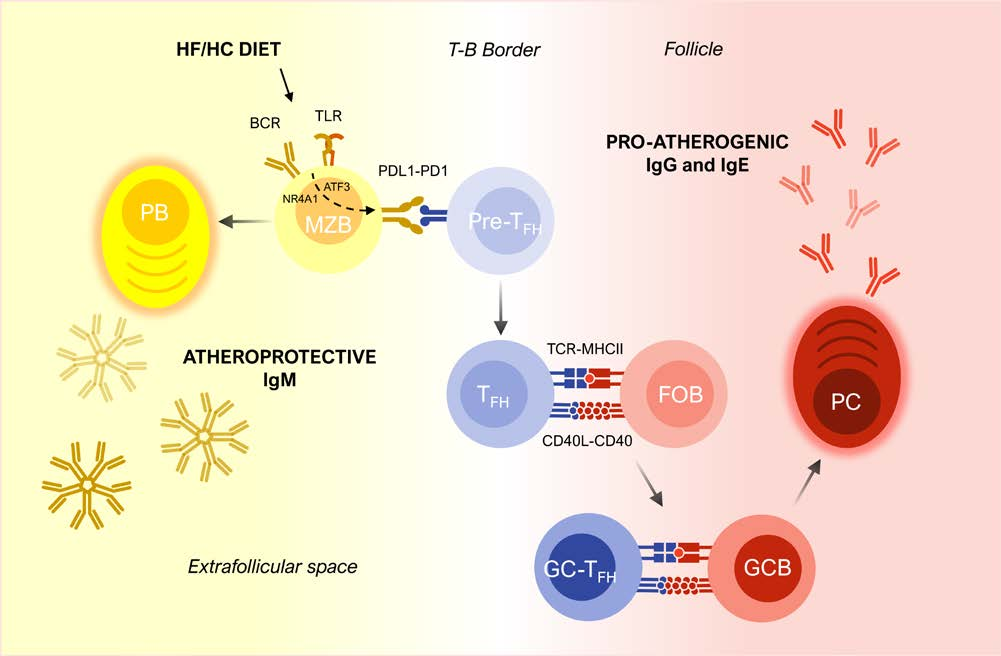
**Splenic B2–T follicular helper (T_FH_) cell interactions in atherosclerosis.** Marginal zone (MZ) B cells sense high-fat/high-cholesterol (HF/HC) diet likely through BCR and TLR signaling upregulating ATF3 and NR4A1 upstream of PDL1. MZB cell PDL1 promotes differentiation of T_FH_ cells at the T-B border and their interaction promotes MZB cell differentiation into atheroprotective IgM-secreting extrafollicular plasmablasts (PBs). T_FH_ cells also interact with follicular B (FOB) cells, through antigen presentation and CD40L costimulation, to seed germinal centers (GCs) and generate IgG-secreting plasma cells (PCs). MHCII indicates major histocompatibility complex class II.

It has been described that T_FH_ cells can also adopt T_H_1, T_H_2, or T_H_17-like phenotypes (T_FH_1, T_FH_2, or T_FH_17 cells), express their effector cytokines, and thereby skew class switching toward particular antibody classes. Recently, there has been identification of another subset, T_FH_13, that expresses GATA3 and BCL6 and secretes high amounts of IL-13.^42^ Their exact roles are starting to be elucidated, but in general, T_FH_1 might be a more potent inducer of high-affinity GC responses specialized in producing IgG2a and IgG2c; T_FH_2 has been associated with IgE, IgG1, and IgG4 class switching; T_FH_13 has been associated to high-affinity anti-allergen anaphylactic IgE^42^; and T_FH_17 has been associated with the production of IgG2a/c and IgG3 autoreactive antibodies.^43^

In atherosclerosis, because T_FH_ cells are expanded upon a high-fat/high-cholesterol diet,^9^ become more proinflammatory in autoimmunity-prone atherosclerotic mice,^44^ and are better known for their role inducing the proatherogenic GC B-cell response and IgG class switching, they were anticipated to be proatherogenic. However, recent experimental atherosclerotic mouse models support a context-dependent role in which their atheroprotective properties are increasingly recognized.

*Ldlr^−/−^* mice transplanted with *Bcl6^−/−^* bone marrow (BM) had increased early and late atherosclerosis that was attributed to proinflammatory macrophage responses^45^ (Table 1). However, these mice will have also lacked T_FH_ cells that could also have contributed to the increased early atherosclerosis. Gaddis et al^41^ showed that *Ldlr^−/−^* mice transplanted with *Cd4*^*Cre/*+^*Bcl6*^*fl/fl*^ BM (mice with no T_FH_) had a modest decrease in late atherosclerosis, suggesting T_FH_ would be proatherogenic although, notably, this was compared with nonlittermate controls. Two consecutive works performed in our laboratory instead indicated an atheroprotective role for T_FH_ in early atherosclerosis, a timepoint where the adaptive immune response has a more prominent role.^47^ In the first work, the absence of MZB cells led to increased atherosclerosis partly due to an accumulation of not fully differentiated T_FH_ cells that resembled pre-T_FH_, pointing to the hypothesis that fully differentiated T_FH_ may be atheroprotective.^9^ Using a similar approach to that of Gaddis et al, we found that *Ldlr^−/−^Rag2*^*−/−*^ (mice with no T cells, to mitigate against recipient T_FH_ cells developing from irradiation-resistant T-cell progenitors) transplanted with *Cd4*^*Cre/+*^*Bcl6*^*fl/fl*^ BM had increased atherosclerosis compared with WT (wild-type) littermates.^25^ T_FH_-cell deficiency led to the accumulation of MZB cells that were unable to become PCs and to a complete abrogation of OSE-specific IgM titers, including T15/E01-IgM, which was previously thought to only be produced by B1 cells in a T cell–independent manner.^7^ Therefore, we concluded that T_FH_ cells were atheroprotective through their role in promoting the production of anti-OSE IgM by MZB cells in an extrafollicular T_FH_-dependent manner. The same results were obtained when blocking IL-18 signaling specifically in T_FH_, which led to reduced T_FH_ cells, MZB cell accumulation, and reduced antibody production, enhancing the development of atherosclerosis. Likewise, Gisterå et al^48^ showed that injection of human LDL-specific murine T cells into human LDL-expressing *Ldlr^−/−^* mice populated the T_FH_-cell compartment and also reduced early atherosclerosis. This was attributed to increased GC activity and anti-OSE IgG and IgM antibodies, which promoted lipoprotein clearance. Conversely, Clement et al^46^ also found that introducing a point mutation (D227K) into Qa1 (a T_FH_-expressed nonclassical MHCI molecule) to block the function of Qa1-restricted CD8^+^ T_REG_s in *Apoe*^*–/–*^ mouse model led to increased atherosclerosis due to T_FH_ accumulation, but whether these were fully differentiated T_FH_ cells was not assessed.

**Table 1.**
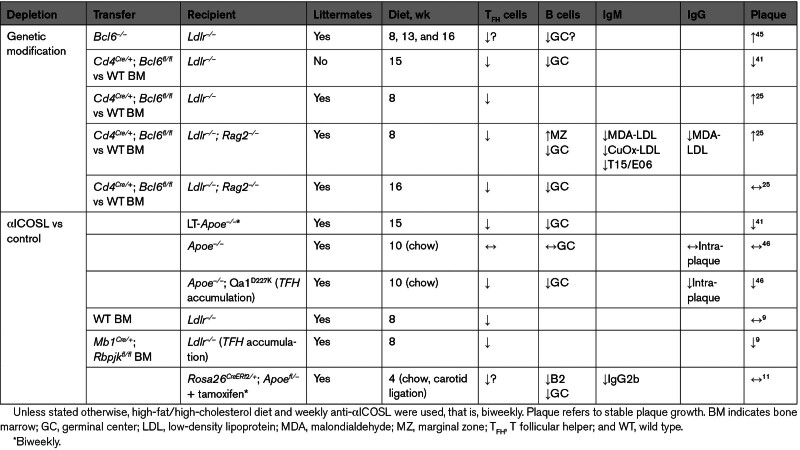
Summary of T_FH_ Cell Depletion Studies in Atherosclerosis

Using another approach to deplete T_FH_ cells with an anti-ICOSL antibody also led to controversial results possibly due to off-target effects. Gaddis et al^41^ showed that administration of anti-ICOSL to *Ldlr^−/−^* mice significantly decreased atherosclerosis, but we and others observed no effect on stable plaques in *Ldlr*^*−/−*9^ and *Apoe*^*–/–*^^11,46^ mice. Anti-ICOSL administration was only protective when there was an accumulation of T_FH_ cells in the absence of MZB cells^9^ or Qa1 CD8^+^ T_REG_s.^46^ Although more circuitous than the ICOS blockade that also depletes T_FH_ cells,^49^ recombinant ICOS-Fc chimera immunization to provoke endogenous ICOS blocking antibody production increased early atherosclerosis in *Apoe^−/−^* mice.^50^ Accordingly, we conclude that the T_FH_-MZB interaction is atheroprotective and is necessary to maintain a balance between the extrafollicular IgM antibody production and GC responses in atherosclerosis (Figure).

Although antibody responses are largely initiated within secondary lymphoid organs, T_FH_ cells may exert functions elsewhere. T_FH_ cells have not been identified within plaques^51^ but can be found, along with GCs and MZB cells, in ATLOs, which form within the adventitia in advanced atherosclerosis.^52^ T_FH_ cells have been detected in increased numbers in ATLOs from patients with abdominal aortic aneurysm^46^ (especially T_FH_1)^53^ and in aged *Apoe*^*–/–*^ mice. T_FH_ cells can recirculate and are readily found in the blood of humans but not of mice. The exact role of circulating T_FH_ and their similarity to T_FH_ or pre-T_FH_ are still unknown.^54^ A few small sized studies have attempted to explore the correlation between circulating T_FH_ cells and cardiovascular disease. In general, increased levels of circulating T_FH_ cells have been associated with increased stenosis severity and coronary artery disease.^55–58^ But further functional studies and larger human populations are needed to establish the significance of circulating T_FH_ cells in cardiovascular disease.

## REGULATION OF T CELL–B CELL INTERACTIONS IN ATHEROSCLEROSIS

Successful T-cell/B-cell interactions often feature 3 signals: antigen presentation, costimulatory/inhibitory molecules, and cytokines. It is important to identify the molecules that might be driving T-cell/B-cell interactions in atherosclerosis to find amenable targets for developing more specific therapeutics. While many of these molecules are expressed by T_FH_ cells, we cannot dismiss the potential relevance of other T-cell subset interactions with B cells.

### Antigen Presentation

#### MHCII

Global MHCII deletion causes congenital CD4^+^ T-cell deficiency; thus, it is not an appropriate model to study T-cell/B-cell interactions.^59^ Using a mixed BM *Ldlr^−/−^* chimera model (Table 2), Tay et al^10^ found B cell–selective MHCII deletion decreased T_FH_ and GC B cells without changing IgM levels, leading to decreased early atherosclerosis in comparison to nonlittermate WT mice. Adoptive transfer of *MHCII*^*−/−*^ B cells into B cell–deficient *Apoe*^*–/–*^ mice also led to decreased atherosclerosis and decreased IgG, but this time, IgM levels were reduced.^33^ Conversely, Williams et al,^60^ using *Cd19*^*Cre/+*^*MHCII*^*fl/fl*^*Ldlr^−/−^* mice and *MHCII*^*fl/fl*^*Ldlr^−/−^* littermates, did not find any effect in atherosclerosis development. These discrepancies could be explained by lack of control littermates, as well as CD19-Cre toxicity and lack of penetrance.^65^ Thus, future experiments using the more efficient *Mb1*^*Cre/+*^ system^66^ and targeting MHCII in different B-cell subsets should shed light on the role of B-cell antigen presentation in atherosclerosis.

**Table 2.**
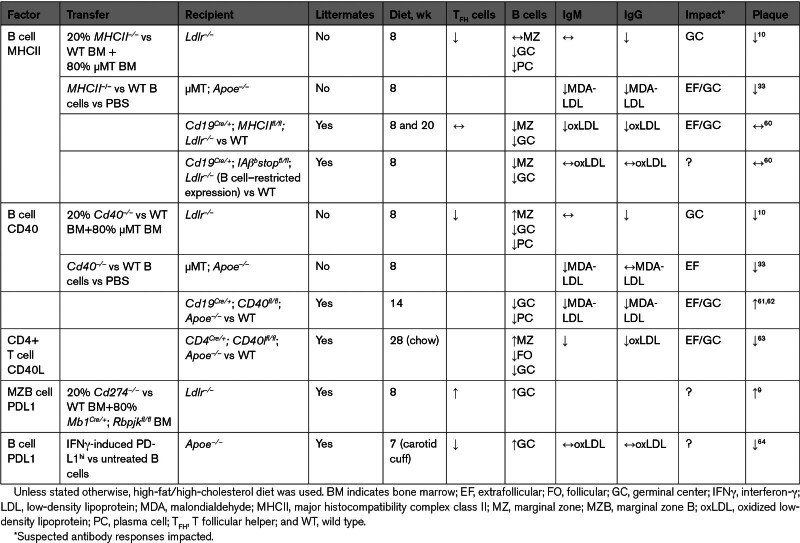
Summary of Cell Type–Selective Evidence Implicating Direct T Cell–B Cell Interaction Molecules in Atherosclerosis

Although most B-cell antigen presentation will occur in secondary lymphoid organs, localized interactions within ATLOs may also regulate plaques in advanced atherosclerosis. An integrated single-cell scRNAseq meta-analysis of human and murine atherosclerotic ATLOs predicted they feature upregulated T-cell/B-cell interactions primarily through MHCII and CD40.^67^ Spatial molecular biology studies should clarify whether these interactions are supporting extrafollicular or GC interactions. scRNAseq also predicted upregulated T-cell/B-cell interactions due to a B-cell MHCII-related gene enrichment in *Apoe*^*–/–*^ mice aorta adventitia in a model of hyperhomocysteinemia-accelerated atherosclerosis.^68^ Mechanistically, homocysteine upregulates the enzyme PKM2, which induces MHCII-related and costimulatory molecule gene expression in B cells, thereby increasing their antigen presentation function. Transfer of PKM2-deficient B cells into anti-CD19/CD20 B cell–depleted *Apoe*^*–/–*^ mice led to fewer MHCII^+^ B cells and lower hyperhomocysteinemia-accelerated atherosclerosis than WT B-cell transfer.

### Costimulatory/Coinhibitory Molecules

#### CD40L-CD40

The primary costimulatory ligand by which T_FH_ cells provide help to activated B cells is CD40L, and ligation of its receptor CD40 in B cells is required for GC responses against T cell–dependent protein antigens.^39^ Using a mixed BM *Ldlr^−/−^* chimera model, Tay et al^10^ found B cell–selective CD40 deletion decreased T_FH_ and GC B cells without changing IgM levels, leading to decreased early atherosclerosis in comparison to nonlittermate WT mice. Adoptive transfer of *CD40*^*−/−*^ B cells into B cell–deficient *Apoe*^*–/–*^ mice also led to decreased atherosclerosis despite decreasing IgM but not affecting IgG levels.^33^ Again, the lack of littermate controls and the difference in models, BM transplant versus B-cell adoptive transfer into B cell–deficient mice, lead to counterintuitive opposing results.^10,33^ Interestingly, preliminary data using B cell–selective CD40 deficiency in *Cd19*^*Cre/+CD40fl/fl*^*Apoe*^*–/–*^ mice versus control littermates showed increased late atherosclerosis due to reduced IgM levels that was associated with the impairment of B1 cells.^61,62^ Global CD40L blockade using antibodies^69^ and CD4^+^ T cell–specific CD40L-deficient mice^63^ was found to decrease late atherosclerosis by various mechanisms involving the innate immune response and smooth muscle cells. In the latter, there was a concomitant GC, IgG, and IgM decrease. Further experiments at earlier timepoints are required to establish the exact role of CD40L-CD40 T-cell/B-cell interactions through targeting them in particular B-cell subsets or in T_FH_ cells.

#### ICOS-ICOSL

As explained above, anti-ICOSL administration to mice did not have an effect on the development of atherosclerosis potentially because of off-target effects.^9,46^ BM transplantation from *Icos*^*–/–*^ donors into *Ldlr*^*–/–*^ recipients led to increased early atherosclerosis attributed to simultaneous loss of T_REG_ numbers and function.^70^ Although T_FH_ cells were not checked in this model, anti-OSE IgG1 and IgG3 were affected but IgM levels were unchanged. Thus, as has been demonstrated previously, ICOS might be important for T_FH_ cell migration to GCs^71^ but not for their extrafollicular localization.

#### PD1-PDL1

Both global PD1 and PD1/PDL1 loss-of-function mouse models have led to increased atherosclerosis through several mechanisms including increased activated T cells in atherosclerotic plaques.^72^ PDL1 is highly expressed in atheroprotective MZB cells and is upregulated in response to a high-fat/high-cholesterol diet.^9^ Specific PDL1 deletion in MZB cells increased early atherosclerosis^9^ and led to the accumulation of T_FH_ cells that were potentially not fully differentiated, as in no MZB cell mice. We have also demonstrated that PDL1 in MZB cells also regulates T_FH_ motility, and, therefore, the PDL1-PD1 interaction may be limiting T_FH_ cells entering GCs. In response to a high-fat/high-cholesterol diet, BCR and TLR signaling pathways activate ATF3^9^ and NR4A1,^73^ leading to PDL1 upregulation. The protective role of PDL1^hi^ B cells was subsequently corroborated through a different mechanism.^64^ Adoptive transfer of IFNγ (interferon-γ)-stimulated B cells that populated both MZ and GC B-cell compartments significantly decreased T_FH_ and PCs, without altering antibody levels and increased T_REG_ cells, leading to reduced atherosclerosis. This indicates IFNγ-stimulated B cells may alter T_FH_ cell numbers but not their function while inducing atheroprotective T_REG_ differentiation. Interestingly, the same group found administration of an agonistic anti-PD1 antibody to *Ldlr*^*–/–*^ mice also reduced early atherosclerosis.^74^ Despite not addressing the impact on T_FH_ cells, which express PD1, they found a significant increase in IgM antibodies, which would fit with the hypothesis that PD1 in T_FH_ favors extrafollicular responses, but further experiments are needed to demonstrate this.

#### GITR-GITRL

B cell–restricted overexpression of another costimulatory ligand, GITRL, in transgenic hCD19-mGITRL *Ldlr*^*–/–*^ BM chimeras also reduced early atherosclerosis.^75^ Although the mechanism is unclear, the authors attributed this to B-cell GITRL directly or indirectly inducing T_REG_ expansion possibly through elevated IL-2 only detected in the thymus and aortas. However, global deletion of the complementary receptor GITR in *Apoe*^*–/–*^ mice was found to also decrease atherosclerosis, with no effect on T_REG_ cell numbers.^76^

#### CD27-CD70

B cell–restricted overexpression of the costimulatory ligand CD70 instead led to increased T effector cells expressing the proatherogenic T_H_1 cytokine IFNγ but, unexpectedly, reduced atherosclerosis in ApoE*3-Leiden mice.^77^ Chronic IFNγ exposure depleted these transgenic B cells and atheroprotective oxLDL antibody titers over time; so this does not offer an explanation. The investigators linked the reduced atherosclerosis to an increase in apoptosis-prone blood monocytes. Hematopoietic deletion of the complementary receptor CD27 in *Apoe*^*–/–*^ mice correspondingly increased atherosclerosis, but this was attributed to a reduction in atheroprotective T_REG_ cells.^78^ While B-cell costimulatory molecule interactions with T_REG_ and effector T cells may be important in atherosclerosis, both GITR and CD27 are also expressed on T_FH_ cells and their role here is largely unexplored.

#### Others

Immune checkpoint inhibitors (ICIs) are being extensively used to target advanced cancer disease successfully, but growing evidence is accumulating that these therapies are associated with increased atherosclerotic plaque development.^79^ These ICIs will also be affecting T-cell/B-cell interactions and impacting the development of atherosclerosis. Apart from the pathways discussed above, others like CD80/CD86-CTLA4, TIM3, and LAG3 are also targeted by ICIs, and they need to be explored specifically in T-cell/B-cell interactions and atherosclerosis.

### Cytokines

Cytokines have been widely implicated in atherosclerosis, but as most of their receptors are ubiquitously expressed, they show pleiotropic effects. For this reason, almost none of the studies have investigated the role of these cytokines at the T-cell/B-cell interaction level. As such, much more studies are required to demonstrate relevant cytokine signaling directly between T and B cells.

#### IL-21

IL-21 is the signature T_FH_ cell cytokine, but it is also produced by other T cells like T_H_17 and natural killer T cells.^80^ It supports activated B-cell proliferation and differentiation into GCs and PCs, but it has many other pleiotropic effects like inhibiting T_REG_ generation. Preliminary data show that pharmacological IL21R blockade in *Ldlr*^*–/–*^ mice reduced early atherosclerosis due to increased T_REG_ activity.^81^ Nevertheless, the effect of IL-21 in T-cell/B-cell interactions and in specific cell subsets remains unexplored.

#### TH1 (IL-12 and IFNγ) and TH2 (IL-5 and IL-4) Cytokines

Traditionally, class-switching antibody isotypes have been associated with different T_H_ cells (ie, IgG2a/2c and T_H_1; IgG1 and T_H_2). Administration of the T_H_1-polarizing cytokine IL-12 to *Apoe^−/−^* mice led to increased IFNγ expression, and so increased atherosclerosis, as well as raised oxLDL-specific IgG2a/c titers that may have contributed partially to their phenotype.^82^ IFNγ has been given a proatherogenic role for skewing the proatherogenic IgG1 to IgG2c in an innate response activator B cell–dependent manner.^83^ Nevertheless, as explained above, others have shown an atheroprotective role upregulating PDL1 in B2 cells.^64^ Thus its role in different B-cell subsets should be addressed.

GC-localized T_FH_ cells eventually express IL-4, which synergizes with IL-21 to promote antibody responses. IL-4 is also produced by T_H_2 cells and may also participate in IgG class switching. Global IL-4 deletion led to reduced atherosclerosis and decreased oxLDL-specific IgG1 antibodies.^84^ T_H_2-derived IL-5 production was also associated with increased secretion of natural T15/E06 IgM from B1 cells.^85^ Thus, hematopoietic IL-5 deficiency in *Ldlr*^*–/–*^ mice led to increased atherosclerosis. Nevertheless, we now know that type 2 innate lymphoid cells are the main source of IL-5.^86^

B cells also express the coinhibitory ligand OX40L, which promotes T_H_2-cell responses. Blockade of OX40L in *Ldlr*^*–/–*^ mice decreased early atherosclerosis.^87,88^ This was linked to reductions in IL-4, thereby reduced oxLDL-specific IgG1, IgE, and a compensatory increase in IL-5, which induced an increase in oxLDL-specific IgM.

#### IL-2

IL-2 is a cytokine secreted by activated CD4^+^ T cells that binds to IL2Rs (IL2RA or CD25; IL2RB or CD122; and IL2RG or CD132) in CD4^+^ and CD8^+^ T cells regulating their function and differentiation. IL-2 promotes T_REG_s but inhibits T_H_17 differentiation.^89^ It has been shown that IL-2 blocks the T_FH_-GC response and consequently IgG antibody production, but IgM levels were not evaluated.^90^ Taking into consideration that in vitro IL-2 with CD40L costimulation induces the formation of IgM-producing PCs,^91^ more studies are needed to explore in vivo other T_FH_ functions. Moreover, recent work from Clatworthy and Mallat laboratories showed that low-dose IL-2 treatment in patients with acute coronary syndrome promoted the generation of IL-10–producing B cells.^92^ Therefore, the role of IL-2 in T-cell/B-cell interactions during atherosclerosis merits further investigation given the potential of using low-dose IL-2 therapy for cardiovascular disease.

## FUTURE DIRECTIONS

Emerging evidence is highlighting the importance of T-cell/B-cell interactions in the development of atherosclerosis. The discovery of new immune cell subsets like T_FH_ with a prominent protective role in atherosclerosis, as well as novel protective properties of B-cell subsets like MZB cells, has opened a new field of research with the aim of understanding the molecular pathways driving T-cell/B-cell interactions that could be targeted to impair atherosclerosis. Furthermore, cardiovascular research could take advantage of ICIs that have already been developed for cancer and use them to specifically target T-cell/B-cell interactions in atherosclerosis and CVD. Using advanced genetic atherosclerotic mouse models selectively targeting T_FH_- and B-cell subsets, we could infer how ICIs are affecting T-cell/B-cell interactions. This new knowledge will be instrumental to develop cell-specific ICI therapies in the future (eg, with the use of encapsulating nanoparticles directed toward specific cells) to treat atherosclerosis.

## ARTICLE INFORMATION

### Sources of Funding

This work was supported by the British Heart Foundation through an Intermediate Basic Science Research Fellowship (FS/20/23/34784) to M. Nus, a Chair of Cardiovascular Medicine (G101517) to Z. Mallat, as well as with project grant to M. Nus and Z. Mallat (PG/22/10898). This work was also supported by the LeDucq Foundation (G107743) and the Horizon 2020 Framework Programme (G106938) to Z. Mallat.

### Disclosures

None.
